# *DYRK1A* – a gene dose paradox

**DOI:** 10.3389/fcell.2026.1963992

**Published:** 2026-10-07

**Authors:** Ante Plećaš, Gillian Gough, Sandra Kunštek, Mirna Babić, Aoife Murray, Dean Nižetić, Ivan Alić

**Affiliations:** 1 Department of Anatomy, Histology and Embryology, Faculty of Veterinary Medicine, University of Zagreb, Zagreb, Croatia; 2 Department of Obstetrics and Gynaecology, Yong Loo Lin School of Medicine, National University of Singapore, Singapore, Singapore; 3 Department of Pathophysiology, Faculty of Veterinary Medicine, University of Zagreb, Zagreb, Croatia; 4 Varaždin General Hospital, Varaždin, Croatia; 5 ARUK-UCL Drug Discovery Institute, Faculty of Brain Sciences, University College London, London, United Kingdom; 6 Faculty of Medicine and Dentistry, Blizard Institute, Queen Mary University of London, London, United Kingdom

**Keywords:** *DYRK1A*, DYRK1A syndrome, down syndrome, iPSCs, disease modelling

## Abstract

The *DYRK1A* (Dual-Specificity Tyrosine Phosphorylation-Regulated Kinase 1A) gene is located on human chromosome 21 (HSA21) within the Down Syndrome Critical Region (DSCR). As a protein kinase, *DYRK1A* is involved in the regulation of the cell cycle, cell growth, transcription, and cellular communication. It also plays essential roles in embryonic and neuronal development, and in disease pathogenesis. Dysregulation of *DYRK1A* has been associated with several human disorders, including *DYRK1A* syndrome, caused by *DYRK1A* haploinsufficiency (i.e., one functional copy of the gene) and Down syndrome (DS), caused by trisomy 21 (i.e., three functional copies of the gene). *DYRK1A* is an extremely dosage-sensitive gene and is considered one of the “Goldilocks” genes, for which both increased and decreased gene dosage can lead to pathological phenotypes. Interestingly, these two syndromes share several overlapping clinical features. This apparent paradox represents/highlights a major gap in our current understanding of *DYRK1A* function and raises important biological questions. In this review, we summarise the most important findings related to both DYRK1A syndrome and Down syndrome, as well as the available human cellular and animal models that provide valuable platforms for future research. To obtain a comprehensive understanding of *DYRK1A* role, it is necessary to integrate data from multiple experimental systems and sources. Such an integrated approach will facilitate the identification of novel therapeutic targets and support the development of treatments that may ultimately advance to clinical trials.

## 1 Introduction

For physiological development from the zygote to the adult organism, an euploid chromosome complement with a species-specific number of genes precisely located on those chromosomes is essential. However, alterations in gene or chromosome copy number may lead to haploinsufficiency, gene overdosage, or chromosomal aneuploidies such as trisomy. Although most of these abnormalities are lethal and lead to early embryonic death, often before placentation, some are compatible with survival depending on the affected gene or chromosome. The resulting phenotypes vary according to the affected genetic region and the extent of gene dosage imbalance. In this review, we summarise the role of the *DYRK1A* (Dual-Specificity Tyrosine Phosphorylation-Regulated Kinase 1A) gene, one of the best-characterised examples of a dosage-sensitive gene which is located on human chromosome 21 (HSA21) within the Down Syndrome Critical Region (DSCR), and discuss its contribution to the pathogenesis of both, DYRK1A syndrome and Down syndrome (DS) (Figure 1). DYRK1A belongs to the CMGC family of protein kinases and primarily regulates cell-cycle progression, cell growth, transcription, and intracellular communication (Varjosalo et al., 2013; Atas-Ozcan et al., 2021; Hammond et al., 2026). In addition, DYRK1A plays essential roles in embryonic development (Johnson et al., 2025), neuronal development including neural progenitor proliferation, neuronal differentiation, dendritic growth, synaptogenesis and brain maturation (Yabut et al., 2010; Tejedor and Hämmerle, 2011; Bellmaine et al., 2017; Pijuan et al., 2022; Cisternas et al., 2025; Courraud et al., 2025) and the pathogenesis of multiple diseases (Guedj et al., 2012; Courraud et al., 2021).

**FIGURE 1:**
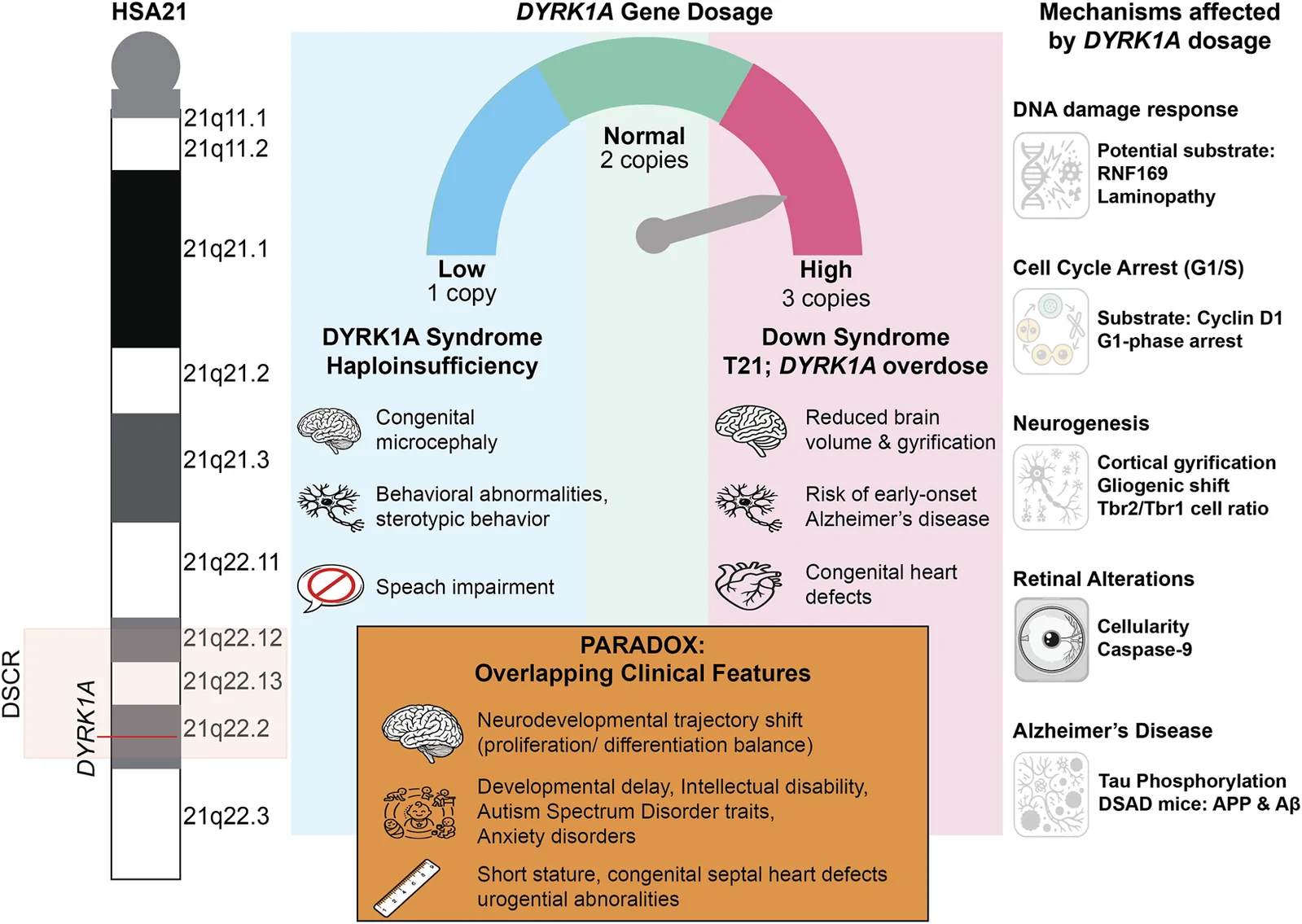
Schematic overview of *DYRK1A* dosage. The *DYRK1A* (Dual-Specificity Tyrosine Phosphorylation-Regulated Kinase 1A) gene is located on human chromosome 21 (HSA21) within the Down Syndrome Critical Region (DSCR) (left column). Haploinsufficiency of *DYRK1A* causes DYRK1A syndrome, whereas trisomy of HSA21 causes Down syndrome (DS). Despite their opposite genetic mechanisms, these two syndromes share several clinical features, highlighted in the orange box (middle column). The right column summarizes the roles of *DYRK1A* in DNA damage response, Cell Cycle Arrest, Neurogenesis, Retinal Alterations and Alzheimer’s disease. The icons in the figure were created with Adobe Illustrator’s generative object prompt. No generative AI was used to generate Figure 1.

## 2 DYRK1A syndrome

DYRK1A syndrome is a rare neurodevelopmental disorder caused by a microdeletion involving *DYRK1A* (21q22.13), resulting in *DYRK1A* haploinsufficiency firstly described less than 20 years ago (Møller et al., 2008; Van Bon et al., 2011). It is characterised by a broad spectrum of clinical features, including congenital microcephaly, moderate to severe intellectual disability, developmental delay, profound speech impairment, short stature, distinctive facial features, behavioural abnormalities including autism spectrum disorder (Kurtz-Nelson et al., 2023), anxiety, stereotypic behaviour and impaired social communication (Ji et al., 2015; Luco et al., 2016; Meissner et al., 2020; Courraud et al., 2021; Pijuan et al., 2022; Glass et al., 2026). Interestingly, despite being caused by *DYRK1A* haploinsufficiency (i.e., loss of one functional copy of *DYRK1A*) DYRK1A syndrome shares several clinical features with Down syndrome (Antonarakis et al., 2020) which is associated with an extra copy of *DYRK1A* due to trisomy 21 (T21) (Figure 1).

DYRK1A syndrome is characterised by intellectual disability, developmental delay, autism spectrum disorder, and seizures. Most of these features are also common in Down syndrome, though the mechanisms may be different. This apparent paradox highlights the importance of precise regulation of *DYRK1A* expression throughout neurodevelopment. Some features are distinctly different, for example, the facial dysmorphism has a different appearance, and microcephaly is not a prominent feature in DS. *DYRK1A* syndrome has also been recorded to clinically mimic other neurodevelopmental conditions, such as Cockayne syndrome (Le May et al., 2025).

Depending on the nature of the causative mutation, the molecular consequences range from loss of protein stability, complete loss of function, or (in some rare cases), even gain of function (summarised in (Courraud et al., 2021)). The effects of missense mutations depend on the protein domain of *DYRK1A* that is affected. For example, some mutations mainly affect the ability of *DYRK1A* to autophosphorylate itself at Tyr321 (Widowati et al., 2018).

## 3 Down syndrome

Down syndrome is the most common viable aneuploidy and is caused by full or partial trisomy of chromosome 21 (T21 or pT21) (Antonarakis, 1998; Papavassiliou et al., 2009; Papavassiliou et al.,2015; Antonarakis et al., 2020). DS is a multisystem disorder characterised by short stature, brachydactyly, hypotonia, a small mouth and ears, congenital heart defects, hypothyroidism, obstructive sleep apnoea, epilepsy, hearing and vision problems, haematological disorders, anxiety disorders, early-onset Alzheimer disease, and autism spectrum disorder (Antonarakis et al., 2020). Interestingly, the majority of these features of DS were also observed in individuals with pT21 (Lyle et al., 2009; Pelleri et al., 2019; Pelleri et al.,2022; Murray et al., 2023), particularly in cases involving duplication of the Down syndrome critical region (DSCR) located on chromosome 21q22.13 (Antonaros et al., 2021) including *DYRK1A* which is one of the most important genes responsible for the neurodevelopmental and cognitive features associated with DS (Ronan et al., 2009; Schnabel et al., 2018; Murray et al., 2023).

## 4 *In vitro* modelling

*In vitro* models have been one of key tools in understanding how altered *DYRK1A* dosage contributes to DS and DYRK1A syndrome phenotypes. Previously, transchromosomic mouse (Canzonetta et al., 2008) as well as mouse and human embryonic stem cells (mESCs and hESCs) were commonly used to study DS-specific phenotypes *in vitro* (Canzonetta et al., 2008; Halevy et al., 2016; Smith et al., 2025). However, the use of hESCs has become more limited due to ethical concerns. An early study using three cell lines (HeLa, U2OS, and HEK-293T) contributed to the identification of molecular targets of *DYRK1A* *.* For example, RNF169 was identified as a *DYRK1A* substrate, revealing an important role of *DYRK1A* in regulation of the cellular DNA damage response (Menon et al., 2019; Roewenstrunk et al., 2019). Moreover, several studies conducted in different cell lines, including both mouse and human cells, have identified *DYRK1A* as a negative regulator of the G_1_/S transition of the cell cycle. *DYRK1A* induces G_1_-phase cell cycle arrest by directly phosphorylating Cyclin D1, thereby promoting its degradation and inhibiting cell cycle progression (Park et al., 2010; Chen et al., 2013; Fernández-Martínez et al., 2015; Vorwerk et al., 2024; Hammond et al., 2026).

The discovery of both mouse (Takahashi and Yamanaka, 2006) and human (Takahashi et al., 2007; Yu et al., 2007) induced pluripotent stem cells (iPSCs) represents one of the most significant breakthroughs in modern science. Over the last two decades several DS hiPSCs have been generated and published (Park et al., 2008; Li et al., 2012; Briggs et al., 2013; Kogut et al., 2018; Ovchinnikov et al., 2018; Yu et al., 2023). Importantly, rare DS isogenic hiPSC lines (Weick et al., 2013; Murray et al., 2015; 2023; Huo et al., 2018; Giffin-Rao et al., 2022) were also generated, providing a valuable biological platform for investigating the role of T21 and/or specific genetic variants in the development of particular phenotypes. Additionally to DS iPSCs, new iPSCs from individuals with DYRK1A syndrome (Ge et al., 2024; Peng et al., 2026) were published.

Recently, significantly increased DNA damage, together with abnormalities of the nuclear envelope labelled with Lamin B1, was demonstrated using two isogenic DS hiPSC models (Murray et al., 2023). Remarkably, this cellular phenotype was rescued by treating with *DYRK1A* inhibitors in hiPSCs and cerebral organoids (Harmine and ID-8), or by CRISPR/Cas9-mediated gene-editing of DYRK1A in the hiPSCs (Murray et al., 2023). Interestingly, reintroduction of *DYRK1A* by plasmid transfection (Rozen et al., 2018) into *DYRK1A* knockout (KO) hiPSCs increased DNA damage (Murray et al., 2023). Importantly, the *in vitro* findings were validated in primary blood cells obtained from an individual with pT21, as well as in human brain and liver tissues collected from individuals with DS and age/sex-matched euploid controls. Finally, reduced *DYRK1A* dosage in pT21 cerebral organoids resulted in a microcephaly-like phenotype and increased cortical gyrification (Murray et al., 2023).

Moreover, it was shown that Leucettinib-21 reduces the activity of *DYRK1A* as well as phosphorylation of the Cyclin D1 in human isogenic iPSCs derived neural progenitor cells (Giffin-Rao et al., 2022; West et al., 2026), while in the cortical neurons along with reduced activity it decreases Tau phosphorylation.

## 5 Animal modelling

HSA21 is the smallest human autosome, containing 235 protein-coding genes, 441 non-coding RNA genes and 188 pseudogenes (Hattori et al., 2000; Howe et al., 2021). The syntenic regions of HSA21 are distributed across three mouse chromosomes: mouse chromosome 16 (Mmu16), mouse chromosome 17 (Mmu17) and mouse chromosome 10 (Mmu10) (Canonica et al., 2024). Mouse *Dyrk1a* is located on Mmu16 (Reeves et al., 1995). Over the past three decades, numerous animal models have been developed and extensively used in research. The pioneering study using *Drosophila mini brain* (mnb) demonstrated altered neural proliferation and reduced brain size (Tejedor et al., 1995) which is the *Drosophila* orthologue of mammalian *DYRK1A* (Tejedor and Hämmerle, 2011).

The Ts65Dn (B6EiC3Sn a/A-Ts (1716) 65Dn/J) mouse has widely used as an animal model of DS (Reeves et al., 1995; Duchon et al., 2011). Due to the overexpression of *Dyrk1a* in Ts65Dn mice, several studies reported increased Tau expression, increased Tau phosphorylation and an altered ratio of 3R-Tau to 4R-Tau, which was deemed to contribute to neurodegenerative changes in the DS nervous system (Liu et al., 2008; Qian et al., 2013; Yin et al., 2017). In addition to Tau pathology, Ts65Dn mice exhibited morphological and functional retinal alterations similar to those observed in individuals with DS, including increased retinal size and cellularity, together with impaired Caspase-9-mediated apoptosis (Laguna et al., 2013). Moreover, the same study showed that another mouse model, transgenic *Dyrk1a* (i.e., transgenic mice carrying one additional copy of the *Dyrk1a* gene), exhibited a similar phenotype (Laguna et al., 2013). *Dyrk1a* dosage normalisation in Ts65Dn mice rescued both the morphological and functional phenotypes. In contrast, *Dyrk1a* ^+/−^ (haploinsufficiency) mice exhibited significantly thinner inner nuclear and inner plexiform layers, together with an increased number of Caspase-9-positive cells. These findings emphasise the role of *Dyrk1a* during retinal development (Laguna et al., 2008; Laguna et al.,2013). The same two mouse strains, Ts65Dn and transgenic *Dyrk1a,* were used in a study that analysed the role of *Dyrk1a* in the cell cycle and differentiation of cortical neurons. Interestingly, the study showed that neocortical radial glial progenitors in transgenic *Dyrk1a* embryos had lower nuclear Cyclin D1 levels than those in the control embryos, along with a longer cell cycle duration. Moreover, the transgenic animals exhibited altered neurogenesis, characterised by a significantly higher number of Tbr2 positive cells and a significantly lower number of Tbr1 positive cells. These changes contribute to a shift towards gliogenesis and an altered proportion of neuronal projections. Furthermore, normalisation of *Dyrk1a* gene dosage restored Cyclin D1 levels and neuronal differentiation in Ts65Dn embryos. Overall, these findings emphasise the role of *Dyrk1a* in the proliferation and differentiation of cortical neurons (Najas et al., 2015). Additional to a gliogenic shift, *Dyrk1a* ^+/−^ mice showed neurological traits associated with the human pathology, such as defective social interactions, stereotypic behaviours and epileptic activity (Pijuan et al., 2022). *Dyrk1a* overexpression in transgenic *Dyrk1a* mice contributes in DS motor phenotypes, especially in motor cholinergic neurons (Arque et al., 2013).

Recently, seven new mouse strains carrying different HSA21-syntenic regions were generated (Lana-Elola et al., 2016). One of these, the Dp1Tyb strain, showed reduced expression of genes involved in mitochondrial respiration and cell proliferation. The same study demonstrated that an increased dosage of *Dyrk1a* was one of the contributing factors to congenital heart defects in Dp1Tyb mice comparable to those observed in individuals with DS (Lana-Elola et al., 2024). In addition to the heart defects, Dp1Tyb mice have wide range of DS specific features, including aberrant erythropoiesis and megakaryopoiesis, reduced bone density, craniofacial changes, altered cardiac function, a pre-diabetic state and deficits in memory, locomotion, hearing and sleep (Lana-Elola et al., 2021). The same mouse strain, Dp1Tyb, exhibited DS-specific craniofacial morphology similar to that observed in individuals with DS, associated with increased Dyrk1a dosage. In addition to these morphological abnormalities, the authors demonstrated impaired neural crest cell proliferation, which is particularly important because many craniofacial structures are derived from the neural crest (Redhead et al., 2023). Enlarged early endosomes and elevated levels of active Rab5 in Dp1Tyb basal forebrain neurons were associated with impaired transport of internalised TrkB from axons to the soma (Blackburn et al., 2026). Finally, the increased GABA inhibition in Dp1Tyb dentate gyrus is caused by overexpression of *Olig1*, *Olig2* and *Dyrk1a* (Hannan et al., 2025).

The most recent Down syndrome-Alzheimer’s disease (DSAD) mouse models with humanized amyloid precursor protein (APP)/amyloid-β (Aβ) have been generated. Those two models exhibit early AD features and show an overexpression of Dyrk1a (Baniowska et al., 2026).

Although different mouse models of DS have been widely used in many studies, several rat models of DS have recently been developed. Compared with mice, rats are larger animals while recapitulating the DS phenotype, making them advantageous for studies that require more complex behavioural, physiological or surgical procedures (Birling et al., 2017; Kazuki et al., 2022; Raza et al., 2026).

## 6 Comparison of model accuracy

Over the last 160 years, since DS was first described by John Langdon Down, and its genetic cause by Jérôme Lejeune, a wide range of animal and human models have been developed to investigate the underlying mechanisms of the disorder. Some of these models are summarised in Table 1. Other animal models also used in DS research are also explained in (Raza et al., 2026). Initially, animal models, particularly mice, were more prevalent in the scientific literature. One of the most widely used models is the Ts65Dn mouse (Reeves et al., 1995), which carries an additional chromosome segment and is trisomic for a region of mouse chromosome Mmu16 that is orthologous to Hsa21. Though very useful for understanding of many DS phenotypes, this model contains an additional set of overdosed genes not from human chromosome 21, making it not entirely specific to the effects of human chromosome 21 triplication. This limitation was not initially recognised. Several other, above mentioned, models have also been used to investigate the role of *DYRK1A* in DS. Interestingly, the Dp1Tyb strain carries an extra copy of approximately 63% of Hsa21-orthologous mouse genes and significantly recapitulates several DS-related phenotypes, including congenital heart defects and altered head morphology (Lana-Elola et al., 2016; Lana-Elola et al.,2024). This makes Dp1Tyb one of the most promising mouse models for studying DS, particularly in the context of *DYRK1A*. Although animal models are indispensable because they enable the investigation of *DYRK1A* in a living organism and therefore allow a more comprehensive understanding of its molecular and pathophysiological mechanisms, one of their major limitations is the lack of complete synteny between Hsa21 and the corresponding rodent chromosomes. In addition, complete knockout of *Dyrk1a* in mice is lethal, which limits the feasibility of studies investigating the consequences of complete *DYRK1A* loss *in vivo*.

**TABLE 1:** Summary of experimental models of both syndromes shown in this manuscript.

Experimental model	Syndrome	DYRK1A copy number	Features	References
Human	DS	T21-3xDYRK1A	Several DS models showed altered expression and downstream signaling of NRSF/REST an alteration in T21 cells throughout differentiation, as a function of *DYRK1A* overdose	Canzonetta et al. (2008); Lepagnol-Bestel et al. (2009); Lu et al. (2011)
	DS	D21-2xDYRK1A T21-3xDYRK1A	Human isogenic T21 iPSCs-derived neurons showed significantly higher amyloid beta expression and DNA damage	Murray et al. (2015)
	DS	D21-2xDYRK1A T21-3xDYRK1A T21-1xDYRK1A T21-0xDYRK1A pT21-3xDYRK1A pT21-1xDYRK1A	Human isogenic T21 iPSCs showed significant changes in DNA damage, shape of nuclear envelope and cerebral organoid size and gyrification under the influence of *DYRK1A*	Murray et al. (2023)
	DYRK1A syndrome	D21-1xDYRK1A	Recently generated iPSCs from a patient with DYRK1A syndrome carrying a heterozygous DYRK1A mutation (c.1024G > T)	Ge et al. (2024)
	DYRK1A syndrome	D21-1xDYRK1A	Recently generated iPSCs from a patient with DYRK1A syndrome carrying a heterozygous DYRK1A mutation (c.1042G>A)	Peng et al. (2026)
Animal	DS -Ts65Dn	T21-3xDyrk1a	One of the most widely used mouse DS models in biomedical research	Reeves et al. (1995)
	DS -Ts65Dn and transgenic model mBACtgDyrk1a	T21-3xDyrk1a T21-2xDyrk1a T21-1xDyrk1a	Trisomy of *Dyrk1a* is necessary and sufficient for abnormal retinal phenotype which were corrected by Dyrk1a gene editing	Laguna et al. (2008) ; Laguna et al. (2013)
	DS -Ts65Dn and transgenic model mBACtgDyrk1a	T21-3xDyrk1a T21-2xDyrk1a T21-1xDyrk1a	Normalisation of *Dyrk1a* gene dosage restored Cyclin D1 levels and neuronal differentiation in these animal models	Najas et al. (2015)
	DS -Dp1Tyb	T21-3xDyrk1a	One of the most recent mouse models which showed congenital heart defects caused by increased dose of *DYRK1A*	Lana-Elola et al. (2016) ; Lana-Elola et al. (2024)
	DS -Dp1Tyb	T21-3xDyrk1a	One of the most recent mouse models which showed specific cranio-facial morphology	Redhead et al. (2023); Otte and Roper, (2024)

In contrast, human models, particularly induced iPSC-derived models, have only been widely used for approximately the last decade. These models enable the investigation of *DYRK1A* in human cells, and advances in CRISPR/Cas9 gene editing, together with the development of *DYRK1A* inhibitors, have facilitated studies in a variety of iPSC-derived cell types (Murray et al., 2023). Particularly promising are recently published iPSC lines derived from individuals with DYRK1A syndrome (Ge et al., 2024; Peng et al., 2026), which provide an opportunity to investigate the effects of *DYRK1A* dysfunction in a human genetic context. However, the major limitation of human iPSC-based models is the lack of complex tissue organisation, regardless of the tissue or cell type being studied. Based on the available evidence, both animal and human models have distinct advantages and limitations and should not be considered mutually exclusive. Rather, an integrated approach combining different model systems is likely to be necessary to obtain a comprehensive understanding of DS and the role of *DYRK1A* in its pathophysiology.

## 7 Discussion

Dysregulation of *DYRK1A* has been linked to numerous human disorders. Intriguingly, increased *DYRK1A* dosage resulting from trisomy 21, contributes to Down syndrome-associated phenotypes, whereas *DYRK1A* haploinsufficiency, causes DYRK1A syndrome. *DYRK1A* is highly expressed during embryonic development and continues to be expressed throughout adulthood, reflecting its involvement in numerous essential biological processes. Because proper physiological function requires a tightly regulated *DYRK1A* dosage, this gene has been described as a “Goldilocks gene” (Johnson et al., 2025), as both increased and decreased expression levels can result in pathological consequences. A comprehensive review of the literature, together with our own findings, indicates that despite representing opposite alterations in gene dosage, *DYRK1A* haploinsufficiency and T21 share several overlapping clinical features. Notably, intellectual disability and autism spectrum disorder are among the most prominent shared phenotypes. Microcephaly represents one of the cardinal features of DYRK1A syndrome; although it is not considered a typical feature of DS, individuals with DS frequently exhibit reduced brain volume with diminished gyrification compared with euploid controls (Pinter et al., 2001; Hamner et al., 2018; Rodrigues et al., 2019; Levman et al., 2024). A similar pattern has also been observed throughout *in vitro* differentiation of human isogenic cortico-striatal spheroids and assembloids (Plećaš et al., 2026). Additional partially overlapping phenotypes include congenital septal heart defects, urogenital abnormalities, short stature, and several other developmental abnormalities. Taken together, these observations emphasize that *DYRK1A* is an exceptionally dosage-sensitive gene that plays essential roles from embryonic development through adulthood. Perhaps the most intriguing aspect is that both reduced and increased *DYRK1A* dosage, haploinsufficiency and T21 respectively, can lead to remarkably similar clinical features. This paradox represents a major gap in our current understanding and raises important biological questions regarding the molecular mechanisms underlying these shared phenotypes. The development of advanced *in vitro* disease models, particularly patient-derived hiPSCs combined with CRISPR/Cas9 genome-editing technology (Murray et al., 2023), now provides an unprecedented opportunity to model these syndromes *in vitro*. The main limitation of *in vitro* models is that they cannot fully reproduce the complexity of whole-organism development due to absence of blood vessels, the heart, and connective tissue; therefore, animal models are necessary for *in vivo* studies. Together, these complementary platforms enable detailed investigation of cellular proliferation, differentiation, neurodevelopmental and neurodegenerative processes, ultimately facilitating the identification of novel therapeutic targets and supporting the development of treatments that may progress to future clinical trials.
